# Comparing CT-guided and fluoroscopic-guided interventions for chronic low back pain management: a randomized trial

**DOI:** 10.1186/s41747-025-00676-w

**Published:** 2026-03-17

**Authors:** Ahmed Awad Bessar, Hazem Abu Zeid Yousef, Abdelrahman A. Omar, Mohammed Salah Mohamed Ahmed Metwaly, Mohamed Medhat Ali Arnaout, Moustafa H. M. Othman

**Affiliations:** 1 Department of Diagnostic Radiology, Faculty of Medicine, Zagazig University, Zagazig, Egypt; 2 Department of Diagnostic Radiology, Faculty of Medicine, Assiut University, Assiut, Egypt; 3 Department of Neurosurgery, Faculty of Medicine, Zagazig University, Zagazig, Egypt

**Keywords:** Fluoroscopy, Injections (epidural), Low back pain, Radiculopathy, Tomography (x-ray computed).

## Abstract

**Objective:**

Low back pain (LBP) is a leading cause of disability, with radicular symptoms often resistant to conservative treatments. While fluoroscopy and computed tomography (CT) play a pivotal role in procedural accuracy, direct comparisons of clinical outcomes remain limited. We compared the efficacy and safety of fluoroscopy—*versus* CT-guided interventions in the management of radicular LBP.

**Materials and methods:**

Adults with chronic LBP were prospectively randomized 1:1 to receive either fluoroscopy-guided or CT-guided interventions. Assessments were conducted at baseline, one week, one month, three months, and six months, and included the visual analog scale (VAS) for pain and the Oswestry disability index (ODI) for functionality. Operative time, radiation exposure, complication rates, and patient satisfaction were evaluated.

**Results:**

Two hundred participants (mean age 51.3 years) were enrolled. Baseline median VAS value was 6.0 in both groups. No significant differences in ODI were observed at any time point. However, VAS values favored fluoroscopy at one (*p* = 0.030), three (*p* = 0.041), and six months (*p* = 0.012). Both groups demonstrated within-group improvements (*p* < 0.001). Radiation exposure (median 352 *versus* 347.5 mGy; *p* = 0.970), operative time (median 22.5 *versus* 23 min; *p* = 0.317), complication rates (96‒99% no complications), and satisfaction levels (≥ 90% satisfied or very satisfied) were similar.

**Conclusion:**

Both fluoroscopy- and CT-guided interventions are safe and effective for managing radicular LBP. Fluoroscopy offers modest advantages in short-term pain relief, while CT provides enhanced anatomical visualization. The choice of imaging guidance should be individualized based on patient characteristics and resource availability.

**Relevance statement:**

Fluoroscopy- and CT-guided interventions offer safe, effective, and tailored treatment options for radicular LBP, supporting personalized, image-guided approaches.

**Key Points:**

- Both fluoroscopy-guided and CT-guided interventions significantly improve chronic radicular LBP, but fluoroscopy provides superior short-term pain relief.
- Fluoroscopy and CT interventions are equally safe, with comparable complication rates, radiation exposure, and procedure durations.
- Selection between fluoroscopy and CT should be based on individual patient needs, procedural goals, and available resources.

**Graphical Abstract:**

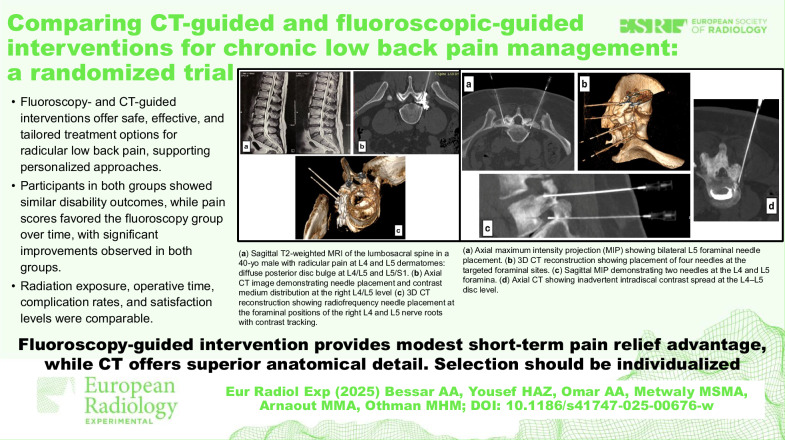

## Background

Low back pain (LBP) is a leading cause of disability globally, posing significant challenges to both individuals and healthcare systems. Its prevalence has increased dramatically over recent decades, driven by aging populations and evolving societal factors [1, 2]. LBP is broadly defined as pain, stiffness, or muscle tension localized below the costal margin and above the inferior gluteal folds, with or without associated radicular symptoms such as sciatica [3, 4].

Chronic LBP, defined as pain persisting for 12 weeks or longer, accounts for a substantial proportion of cases and often fails to respond to conservative management, necessitating advanced interventional strategies [5, 6]. Radicular LBP, which results from nerve root irritation or compression, presents as pain radiating along specific dermatomes and typically requires precise interventions targeting the dorsal root ganglion [7–9].

Advances in imaging technologies—particularly fluoroscopy and computed tomography (CT)—have revolutionized interventional management of radicular LBP. These modalities facilitate accurate needle placement, thereby minimizing complications such as intravascular or intrathecal injection and reducing the risk of injury to critical structures [10, 11]. Among available procedures, transforaminal epidural steroid injections and pulsed radiofrequency (RF) have gained prominence for their effectiveness in alleviating radicular pain, enhancing functional outcomes, and improving patient satisfaction, while surgical intervention remains a curative option in select cases, such as expelled herniated disks [12, 13].

Transforaminal epidural steroid injections, a minimally invasive procedure, involve the injection of corticosteroids and local anesthetics around the affected nerve roots to reduce inflammation and relieve pain. This approach is particularly valued for its rapid onset, lower trauma, and reduced complication rates compared to surgical alternatives [14, 15]. In contrast, pulsed RF employs RF energy to modulate neural activity around the dorsal root ganglion, offering sustained pain relief with minimal tissue damage [16]. When used in combination, these modalities have demonstrated significant improvements in pain scores, functional outcomes, and overall quality of life for patients with radicular LBP [12, 17, 18].

Despite the growing adoption of imaging-guided techniques, the comparative benefits and outcomes of CT-guided *versus* fluoroscopy-guided procedures remain insufficiently studied. Both modalities offer distinct advantages: fluoroscopy enables real-time visualization for accurate needle placement, while CT provides superior anatomical resolution for enhanced procedural precision. A comprehensive understanding of their relative efficacy, technical considerations, and patient-reported outcomes is essential for informed clinical decision-making.

This study aims to address this gap by evaluating the efficacy, safety, and patient satisfaction associated with CT-guided *versus* fluoroscopy-guided interventions in the treatment of radicular LBP. The findings aim to support evidence-based practice and guide clinicians in selecting the most appropriate, minimally invasive treatment tailored to individual patient needs.

## Materials and methods

### Study design and setting

This prospective, non-blinded interventional clinical trial was conducted on patients with chronic radicular LBP associated with sciatica at the Radiodiagnosis Departments of Assiut and Zagazig University Hospitals over a 12-month period. The study adhered to the Strengthening the Reporting of Observational Studies in Epidemiology‒STROBE checklist [19] and received ethical approval from the institutional review boards (IRB number: 17200713). It was conducted in alignment with the Declaration of Helsinki (1964, most recently revised October 2013) [20]. Written informed consent was obtained from all participants prior to enrollment. All patients were initially evaluated in a multidisciplinary setting, including neurosurgical consultation, and those with surgically remediable pathologies (*e.g*., sequestrated hernia) were excluded.

### Eligibility criteria

Participants included in the study met the following conditions:

1. adults aged 18 years or older;
2. chronic radicular LBP with unilateral or bilateral sciatica lasting more than six months, unresponsive to medical treatment for at least six weeks;
3. Pain exacerbated by forward flexion, accompanied by leg numbness and tingling;
4. Visual analog scale (VAS) score of ≥ 5;
5. Positive straight leg raise test with calf and leg pain, without motor deficits;
6. Magnetic resonance imaging (MRI) showing disc pathology at one or more of the three lowest lumbar levels, with no other significant abnormalities;

Exclusion criteria included progressive motor neurological deficits; more than three degenerated disks on MRI; advanced disc pathology (sequestration, extrusion, or spondylolisthesis); moderate to severe spinal stenosis; prior lumbar surgery at the target level; spinal deformities or fractures; systemic infections; uncontrolled comorbidities; pregnancy; and red flag symptoms such as cauda equina syndrome or persistent fever.

### Study workflow and procedural details

**Fig. 1:**
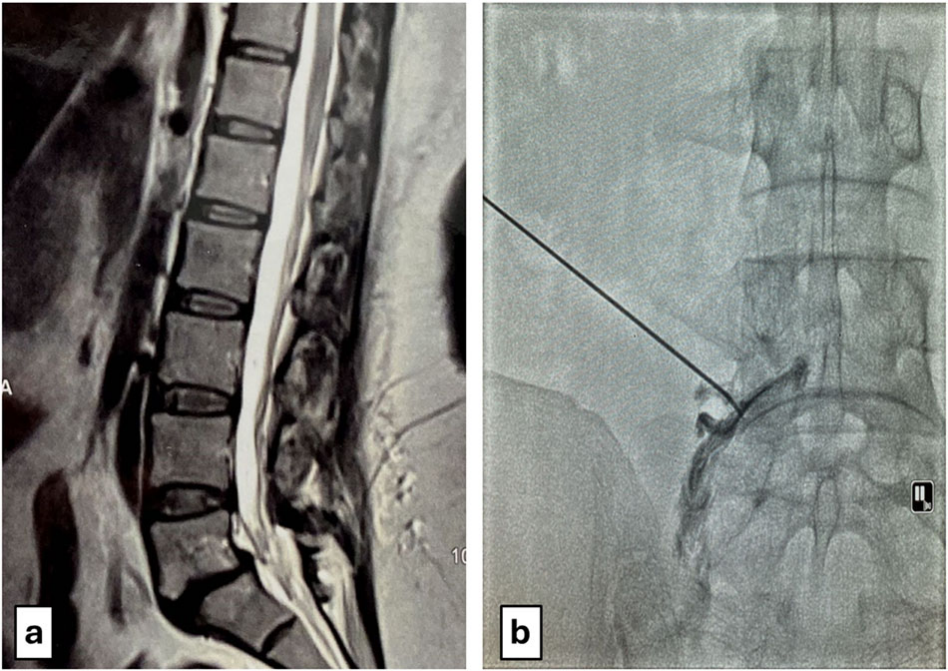
**a** Sagittal T2-weighted MRI of the lumbosacral spine showing diffuse posterior disc bulge at the L5‒S1 level in a 45-year-old female, with mild anterolisthesis and grade II end plate changes. Clinical examination revealed LBP with sciatica and referred pain in the L5 dermatome. **b** Fluoroscopic-guided image demonstrating contrast distribution along the left L5 nerve root sheath

**Fig. 2:**
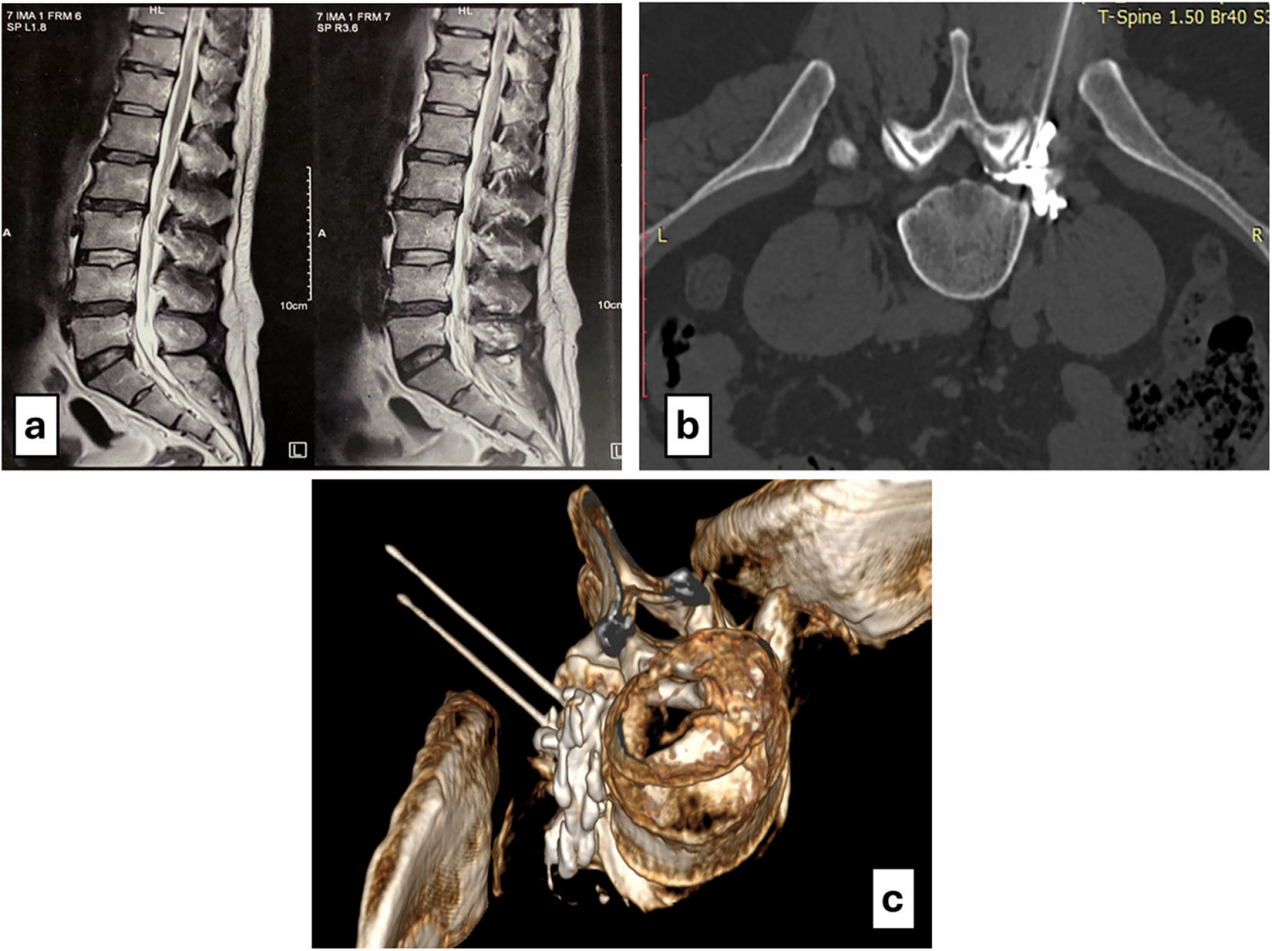
**a** Sagittal T2-weighted MRI of the lumbosacral spine in a 40-year-old male with diffuse posterior disc bulge at the L4–L5 and L5–S1 levels. Clinical examination revealed radicular pain in the L4 and L5 dermatomes. **b** Axial CT image demonstrating needle placement and contrast medium distribution at the right L4/L5 level. **c** 3D CT reconstruction showing RF needle placement at the foraminal positions of the right L4 and L5 nerve roots with contrast tracking

**Fig. 3:**
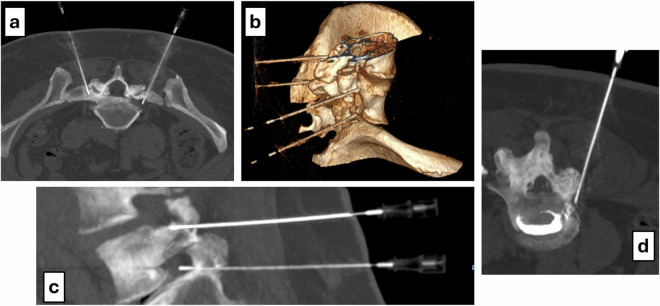
**a** Axial maximum intensity projection (MIP) showing bilateral L5 foraminal needle placement. **b** Three-dimensional CT reconstruction showing placement of four needles at the targeted foraminal sites. **c** Sagittal MIP image demonstrating two needles at the L4 and L5 foramina. **d** Axial CT showing inadvertent intradiscal contrast spread at the L4–L5 disc level

Participants were randomized in a 1:1 ratio using a computer-generated sequence into group A (fluoroscopy-guided) or group B (CT-guided). Stratification within each group was based on the number of puncture points (one to six) for bilateral foraminal injections at the three lowest lumbar levels. Pulsed RF ablation was performed at L3‒L4, L4‒L5, and L5‒S1 levels, with sacroiliac joint injections added based on clinical findings. Procedural steps are illustrated in Figs. 1–3.

All procedures were performed with the patient in the prone position under strict aseptic technique. After disinfection and local anesthesia with 2% lidocaine, an 18‒20-gauge, 10–15 cm RF needle was inserted under the assigned imaging guidance.

All fluoroscopy-guided interventions were performed with patients in the prone position on the procedure table. A paramedian transforaminal approach was used for needle placement, targeting the dorsal root ganglion. Under real-time fluoroscopic guidance, 1–2 mL of nonionic iodinated contrast was injected to confirm accurate needle positioning and rule out intravascular or intrathecal spread. Procedures were conducted using a Philips Azurion cathlab fluoroscopy system (Philips Healthcare) at a frame rate of 3 frames/s.

CT-guided procedures were similarly performed with patients in the prone position on the CT table. A transforaminal approach was used, and multiplanar reconstructions guided the needle trajectory. After needle placement, 1–2 mL of iodinated contrast was injected to confirm correct epidural or foraminal distribution. Procedures were conducted using a Siemens SOMATOM GO. Now, a 16-slice CT scanner (Siemens Healthineers). All infiltrations were performed by two consultant interventional radiologists with more than 10 years of experience in spine interventions.

For the post-RF drug regimen, a mixture of 1–2 mL of 1% lidocaine and 8 mg dexamethasone was injected at each target level. In cases involving sacroiliac joint injection, 2 mL of 0.25% bupivacaine with 8 mg dexamethasone was used. Methylprednisolone acetate was added as needed, not exceeding 80 mg per patient.

For RFA, pulsed RF was delivered using an Abbott RF generator with settings of 42 °C, 2 Hz frequency, and 20 ms pulse width for a total of 300 s per site. Sensory and motor stimulation tests were performed prior to RF delivery to confirm accurate needle placement.

Radiation dosimetry was recorded for all procedures. In the fluoroscopy group, dose area product‒DAP (mGy·cm²) was obtained directly from the cathlab system. In the CT group, volume CT dose index‒CTDIvol (mGy) and dose-length product‒DLP (mGy·cm) were automatically generated by the CT scanner. A low-dose protocol (120 kVp, 50–70 mAs, with automatic dose modulation) was applied in all cases.

Patients were informed of the imaging guidance modality on the day of the procedure. All procedures were performed with patients in the prone position, and operative time was recorded as the interval between skin localization and removal of the RF needle, excluding room-in and anesthesia preparation time. For patients with multiple punctures, the median procedural time per puncture was calculated by dividing the total duration by the number of punctures.

Prior to intervention, a comprehensive clinical evaluation was conducted, including pain history (severity, location, aggravating and relieving factors), VAS, Oswestry Disability Index (ODI), lumbosacral X-rays to exclude structural deformities, and MRI to confirm discogenic pain.

### Postprocedural care and follow-up

After the procedure, patients were observed in the prone position for 1–2 h. If no neurological deficits or complications were noted, they were discharged the same day. As per institutional protocol, patients were advised to observe relative bed rest for 24 h, after which they could gradually resume normal activities, including back muscle strengthening exercises.

Patients received 1 g of paracetamol intravenously as needed for mild pain. Non-steroidal anti-inflammatory drug (NSAIDs)—diclofenac sodium 75 mg intramuscularly or equivalent oral dose—were prescribed if pain persisted.

From the second day onward, strenuous activity was restricted for three months. Follow-up assessments were performed at one week, and at 1-, 3-, and 6-month post-procedure. Each follow-up included evaluation of VAS, ODI, and patient satisfaction, which was assessed via a Likert scale ranging from “strongly satisfied” to “very unsatisfied” [21]. Drug usage and any procedure-related complications were also recorded.

Patients were discharged with oral NSAIDs (diclofenac 50 mg twice daily or equivalent) for breakthrough pain, with instructions to taper the dose as symptoms improved. Opioids were reserved for refractory cases: if required, tramadol (50–100 mg/day) or oral morphine equivalents (10–20 mg/day) were prescribed.

### Outcome measures

Primary outcomes included changes in VAS and ODI scores, complication rates, and patient satisfaction. Secondary outcomes included opioid consumption (expressed in oral morphine equivalents), radiation exposure, and median procedural time.

### Statistical analysis

Statistical analysis was performed using IBM SPSS Statistics for Windows, version 20.0 (IBM Corp). Categorical variables were expressed as counts and percentages, while continuous variables were summarized using mean, standard deviation, median, and interquartile range (IQR). Normality of continuous variables was assessed using the Kolmogorov-Smirnov test, with *p* < 0.05 considered significant. Comparisons between groups were conducted using the χ² test for categorical variables, Student *t*-test for normally distributed continuous variables, and the Mann–Whitney *U*-test for non-normally distributed data. Repeated measures (*e.g*, VAS and ODI across timepoints) were analyzed using the Friedman test, with Dunn’s post hoc pairwise comparisons applied when appropriate.

## Results

### Demographic and baseline characteristics

The mean age of participants was 51.29 ± 8.77 years (mean ± standard deviation) in group A (fluoroscopy-guided) and 52.71 ± 10.11 years in group B (CT-guided), without a significant difference (*p* = 0.290). Gender distribution was also similar between groups (*p* = 0.776). Assessment of nerve root laterality at the L3, L4, and L5 levels revealed no significant group differences in unilateral or bilateral involvement (*p* = 0.756, *p* = 0.672, and *p* = 0.754, respectively), as shown in Table 1.

**Table 1:** Demographic and baseline characteristics

Characteristic	Group A (*n* = 100)	Group B (*n* = 100)	*p*-value^*^
Age (years)			
Mean ± standard deviation	51.29 ± 8.77	52.71 ± 10.11	0.290
Median (IQR)	50.0 (45.0–58.0)	54.0 (45.0–60.0)	
Gender			
Male	46 (46.0%)	44 (44.0%)	0.776^χ²^
Female	54 (54.0%)	56 (56.0%)	
L3 root laterality			0.756^χ²^
No	94 (94.0%)	95 (95.0%)	
Bilateral	6 (6.0%)	5 (5.0%)	
L4 root laterality			0.672^χ²^
No	21 (21.0%)	20 (20.0%)	
Unilateral	43 (43.0%)	38 (38.0%)	
Bilateral	36 (36.0%)	42 (42.0%)	
L5 root laterality			0.754^χ²^
No	10 (10.0%)	12 (12.0%)	
Unilateral	47 (47.0%)	42 (42.0%)	
Bilateral	43 (43.0%)	46 (46.0%)	

*Group A* Fluoroscopy-guided injection, *Group B* CT-guided injections, *IQR* Interquartile range

^*^ χ² test

The distribution of nerve root injections was similar, with most patients receiving one to two injections (73.0% in group A *versus* 70.0% in group B; *p* = 0.778). Bilateral injections were most frequent in both groups (42.0% in group A *versus* 44.0% in group B). Laterality types (none, right-sided, left-sided, bilateral) were also not significantly different (*p* = 0.880), as detailed in Table 2.

**Table 2:** Comparison between the two studied groups according to the roots injected and the side of injection

Variable	Group A (*n* = 100)	Group B (*n* = 100)	*p*-value^*^
Roots injected			0.778
1 or 2	73 (73.0%)	70 (70.0%)	
3 or 4	21 (21.0%)	25 (25.0%)	
5 or 6	6 (6.0%)	5 (5.0%)	
Side of injection (SI)			0.880
No	22 (22.0%)	25 (25.0%)	
Only right	21 (21.0%)	19 (19.0%)	
Only left	15 (15.0%)	12 (12.0%)	
Bilateral	42 (42.0%)	44 (44.0%)	

*SI* Sacroiliac joint, *Group A* Fluoroscopy-guided injection, *Group B* CT-guided injections

^*^ χ² test

### ODI

**Fig. 4:**
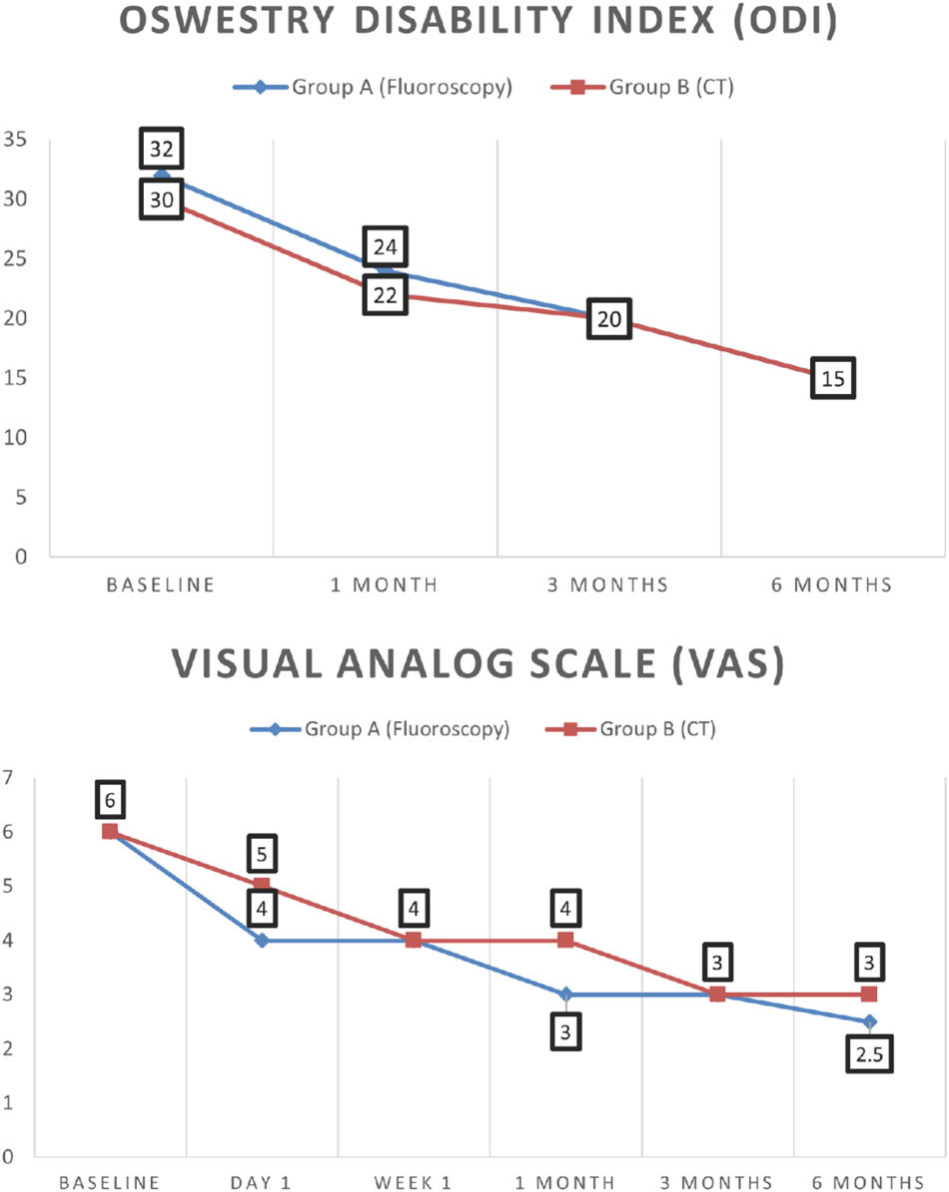
Comparison of ODI and VAS scores over time between the two study groups

Baseline ODI scores were slightly higher in group A (median 32.0) compared to group B (median 30.0), but the difference was not statistically significant (*p* = 0.206). At 1-, 3-, and 6-month post-intervention, both groups demonstrated significant within-group improvements in ODI scores (*p* < 0.001 for each group). However, no significant differences were observed between groups at any time point (all *p* > 0.05), as shown in Table 3 and Fig. 4.

**Table 3:** Comparison between the two studied groups according to ODI and VAS

Variable	Group A (*n* = 100)	Group B (*n* = 100)	*p*-value^*^
ODI			
Before	32.0 (25.0–40.0)	30.0 (24.0–35.0)	0.206
1 month	24.0 (20.0–30.0)	22.0 (17.0–30.0)	0.654
3 months	20.0 (15.0–24.0)	20.0 (15.0–24.0)	0.688
6 months	15.0 (12.0–20.0)	15.0 (12.0–20.50)	0.775
Fr (ODI)	251.976	246.220	
p0 (ODI)	< 0.001^*^	< 0.001^*^	
VAS			
Before	6.0 (5.0–8.0)	6.0 (5.0–8.0)	0.500
1 day	4.0 (4.0–5.0)	5.0 (4.0–6.0)	0.404
1 week	4.0 (3.0–5.0)	4.0 (3.0–5.0)	0.084
1 month	3.0 (2.0–4.0)	4.0 (3.0–5.0)	0.030
3 months	3.0 (2.0–4.0)	3.0 (2.0–4.0)	0.041
6 months	2.50 (2.0–4.0)	3.0 (2.0–4.0)	0.012
Fr (VAS)	419.176	384.408	
p0 (VAS)	< 0.001^*^	< 0.001^*^	

Data are given as median (IQR)

*Group A* Fluoroscopy-guided injection, *Group B* CT-guided injections, *Fr* Friedman test, *p0* p-value for comparing different study periods using post hoc Dunn’s test

^*^ Mann–Whitney *U*-test

### VAS

Baseline VAS scores were similar in both groups, with no significant difference (*p* = 0.5). Both groups experienced significant reductions in VAS scores over time (*p* < 0.001 within each group). However, group A showed significantly greater pain reduction at 1 month (*p* = 0.030), 3 months (*p* = 0.041), and 6 months (*p* = 0.012) compared to group B, as presented in Table 3 and Fig. 4.

### Radiation dose and operative time

Median radiation doses were comparable between group A (352.0 mGy) and group B (347.5 mGy), with no statistically significant difference (*p* = 0.970). Median operative times were also similar: 22.50 min in group A and 23.0 min in group B (*p* = 0.317), as shown in Table 4.

**Table 4:** Radiation dose and operative time

Variable	Group A (*n* = 100)	Group B (*n* = 100)	*p*-value^*^
Radiation dose (mGy)	352.0 (270.5–415.0)	347.5 (265.0–388.5)	0.970
Operative time (min)	22.50 (19.0–30.0)	23.0 (19.0–30.0)	0.317

Data are given as median (IQR)

*Group A* Fluoroscopy-guided injection, *Group B* CT-guided injections

^*^ Mann–Whitney *U*-test

### Safety, *c*ontrast *e*xtravasation, and *m*edication *u*se

As shown in Table 5, both groups experienced low complication rates, with no significant difference (*p* = 0.385). In group A (*n* = 100), one patient (1.0%) developed an abscess; no cases of discitis/neuritis or hematoma were reported. In group B (*n* = 100), one patient (1.0%) developed an abscess, one (1.0%) had discitis/neuritis, and two (2.0%) experienced hematoma. Overall, 99% of group A and 96% of group B had no complications. Contrast extravasation occurred in 5.0% of patients in both groups, with no difference between group A and group B (*p* = 1.000).

**Table 5:** Safety, contrast extravasation, and medication use

Variable	Group A (*n* = 100)	Group B (*n* = 100)	*p*-value
Complications			0.385^*^
No	99 (99.0%)	96 (96.0%)	
Abscess	1 (1.0%)	1 (1.0%)	
Discitis and neuritis	0 (0.0%)	1 (1.0%)	
Hematoma	0 (0.0%)	2 (2.0%)	
Contrast Extravasation			1.000^*^
Negative	95 (95.0%)	95 (95.0%)	
Positive	5 (5.0%)	5 (5.0%)	
Reduction of NSAIDs (%), median (IQR)	70.0 (60.0–80.0)	70.0 (50.0–80.0)	0.524^**^
Morphine equivalent			0.407^*^
Pre (0 mg)	96 (96.0%)	98 (98.0%)	
Pre (10 mg)	4 (4.0%)	2 (2.0%)	
Morphine equivalent (1 month)			0.316^*^
Pre (0 mg)	100 (100.0%)	99 (99.0%)	
Pre (20 mg)	0 (0.0%)	1 (1.0%)	

*Group A* Fluoroscopy-guided injection, *Group B* CT-guided injections*, IQR* Interquartile range, *NSAID* Non-steroid anti-inflammatry drug

^*^ χ² test

^**^ Mann–Whitne&&y *U*-test

NSAID use declined similarly in both groups: a median reduction of 70.0% (IQR: 60.0–80.0) in group A and 70.0% (IQR: 50.0–80.0) in group B (*p* = 0.524). Opioid use was minimal: at baseline, 4.0% of group A and 2.0% of group B used 10 mg morphine. At one month, no patients in group A and 1.0% in group B used 20 mg morphine. These differences were not statistically significant (*p* = 0.407 and *p* = 0.316, respectively), as shown in Table 5.

### Satisfaction with procedure and results

Most patients were either satisfied or very satisfied with the procedure. In group A, 63.0% were satisfied and 27.0% very satisfied; in group B, 65.0% were satisfied and 25.0% very satisfied, with no significant difference (*p* = 0.870). Satisfaction with the outcome was also high: 49.0% of group A and 58.0% of group B were satisfied, while 45.0% of group A and 35.0% of group B were very satisfied. These differences were not statistically significant (*p* = 0.552), as shown in Table 6.

**Table 6:** Comparison of procedures and results satisfaction

Variable	Group A (*n* = 100)	Group B (*n* = 100)	*p*-value^*^
Satisfaction with the procedure			0.870
Very unsatisfied	0 (0.0%)	1 (1.0%)	
Unsatisfied	6 (6.0%)	6 (6.0%)	
Satisfied	63 (63.0%)	65 (65.0%)	
Very satisfied	27 (27.0%)	25 (25.0%)	
Satisfaction with results			0.552
Very unsatisfied	1 (1.0%)	1 (1.0%)	
Unsatisfied	5 (5.0%)	6 (6.0%)	
Satisfied	49 (49.0%)	58 (58.0%)	
Very satisfied	45 (45.0%)	35 (35.0%)	

*Group A* Fluoroscopy-guided injection, *Group B* CT-guided injections

^*^ χ² test

## Discussion

This study demonstrated that both CT-guided and fluoroscopy-guided interventions are safe and effective for treating radicular LBP. Pain and disability, as measured by VAS and ODI, improved significantly in both groups, consistent with previous findings on imaging-guided injections for radicular pain [22]. Although both groups showed comparable long-term outcomes, the fluoroscopy-guided group achieved slightly greater short-term pain reductions at 1, 3, and 6 months. This short-term advantage may be attributed to the real-time visualization provided by fluoroscopy, which facilitates precise needle placement and optimal targeting of inflamed nerve roots. Another potential explanation is the shorter procedural time observed with fluoroscopy, which could reduce patient discomfort and procedural stress, possibly influencing early pain perception. Additionally, fluoroscopy enables continuous monitoring of pain provocation during needle advancement, allowing for more customized drug delivery. These factors may collectively explain the modest but consistent short-term pain relief advantage associated with fluoroscopy, despite the overall similarity in long-term outcomes between modalities [23].

While a small proportion of patients used morphine, its distribution was balanced between groups, and the absolute numbers were too low to impact group-level VAS outcomes. Furthermore, statistical comparisons revealed no significant differences in NSAID or opioid use, indicating that post-procedural medication patterns were unlikely to account for the observed differences in pain relief.

Both interventions demonstrated low complication rates, with no significant between-group differences, supporting prior evidence on the safety of imaging-guided procedures [10, 24–26]. Radiation exposure and procedure duration were also comparable across groups, reinforcing findings that protocol optimization can mitigate radiation concerns associated with CT guidance [27]. The high patient satisfaction rates in both groups further support the clinical utility of these techniques for managing chronic radicular symptoms.

These results align with previous studies validating transforaminal epidural steroid injections and pulsed RF for radicular LBP management. Earlier research highlighted the efficacy of imaging-guided transforaminal epidural steroid injections in reducing pain and improving function [22], while others emphasized the precision and safety of both fluoroscopy- and CT-guided injections [25]. Fluoroscopy excels in real-time monitoring of needle trajectory and injectate spread [28], whereas CT offers superior anatomical resolution, particularly beneficial in anatomically complex cases [27].

Despite the growing use of both modalities, direct comparative studies remain limited. This study demonstrates equivalent long-term efficacy and safety, with fluoroscopy providing a short-term analgesic advantage, thereby contributing evidence to the evolving landscape of interventional spine care.

This study design, involving both fluoroscopic and CT-guided cohorts, enabled a direct comparison of efficacy, safety, radiation exposure, and patient satisfaction, providing a comprehensive, clinically relevant perspective. However, several limitations must be acknowledged. The lack of blinding introduces potential observer bias, particularly for subjective outcomes. As a single-center study, generalizability may be limited due to institutional practice patterns. Additionally, the six-month follow-up period may not fully capture long-term outcomes or rare adverse events. Larger, multicenter trials with extended follow-up are needed to further validate these findings and assess infrequent complications.

From a practical perspective, fluoroscopy- and CT-guided techniques should be considered complementary rather than competing. The choice of modality can be based on patient-specific factors, local resources, and procedural objectives. Fluoroscopy is advantageous when real-time feedback and rapid adjustments are essential. In challenging anatomical scenarios, angiographic systems with cone-beam CT capability provide a unique hybrid solution, enabling both fluoroscopic real-time feedback and high-resolution cross-sectional imaging within the same session. This integration may overcome limitations of either modality alone and is of particular value in complex neurointerventional and spine procedures [29].

Ultimately, a patient-centered approach should guide modality selection—favoring fluoroscopy when accessibility and real-time guidance are key, and opting for CT when anatomical precision is paramount. In either case, the primary goal remains the same: delivering effective, safe, and individualized care for patients with radicular LBP.

Future research should focus on the main key areas. First, cost-effectiveness and accessibility analyses are necessary to understand how resource constraints influence modality selection. Second, longer-term follow-up is warranted to assess the durability of symptom relief and late-onset adverse events. Third, incorporating more patient-reported outcomes, including quality of life and psychological well-being, could help tailor interventional strategies to individual preferences and needs.

In conclusion, both fluoroscopy- and CT-guided interventions are reliable, safe, and well-tolerated options for managing radicular LBP. Fluoroscopy offers real-time visualization and more immediate pain relief, while CT provides superior anatomical resolution, particularly in patients with complex or altered spinal anatomy. Rather than being viewed as competing techniques, these modalities should be considered complementary, with selection tailored to each patient’s clinical presentation, procedural objectives, and resource availability.

In challenging cases, hybrid angiographic platforms equipped with cone-beam CT can integrate real-time fluoroscopic guidance with three-dimensional imaging, thereby overcoming the limitations of either modality alone. While such integration may incur higher costs, it offers the potential for maximized precision, enhanced safety, and improved patient-centered outcomes. When feasible, this combined approach expands the interventional radiologist’s capabilities for individualized spine care.

## Data Availability

All data relevant to the study are included in the article or uploaded as supplemental information. The raw data supporting this study’s findings are available from the corresponding author upon reasonable request.

## Abbreviations

CT: Computed tomography
IQR: Interquartile range
LBP: Low back pain
MRI: Magnetic resonance imaging
NSAID: Non-steroidal anti-inflammatory drug
ODI: Oswestry disability index
RF: Radiofrequency
VAS: Visual analog scale

## References

[1] Buchbinder R, van Tulder M, Öberg B et al (2018) Low back pain: a call for action. Lancet 391:2384–2388. 10.1016/S0140-6736(18)30488-4 29573871 10.1016/S0140-6736(18)30488-4

[2] Nottidge BA, Odole AC, Odunaiya NA et al (2019) Development and structural validity of a Nigerian culture- and environment-friendly low back pain outcome measure: Ibadan low back pain disability questionnaire. Ghana Med J 53:126–134. 10.4314/gmj.v53i2.7 31481808 10.4314/gmj.v53i2.7 PMC6697772

[3] Hartvigsen J, Hancock MJ, Kongsted A et al (2018) What low back pain is and why we need to pay attention. Lancet 391:2356–2367. 10.1016/S0140-6736(18)30480-X 29573870 10.1016/S0140-6736(18)30480-X

[4] Napoli A, Alfieri G, Scipione R et al (2020) Pulsed radiofrequency for low-back pain and sciatica. Expert Rev Med Devices. 10.1080/17434440.2020.1719828 10.1080/17434440.2020.1719828 31973587

[5] Chou R (2021) Low back pain. Ann Intern Med 174:ITC113–ITC128. 10.7326/AITC202108170 34370518 10.7326/AITC202108170

[6] Jinnouchi H, Matsudaira K, Kitamura A et al (2019) Effects of low-dose therapist-led self-exercise education on the management of chronic low back pain: protocol for a community-based, randomized, 6-month parallel-group study. Spine Surg Relat Res 3:377–384. 10.22603/ssrr.2019-0005 31768459 10.22603/ssrr.2019-0005 PMC6834468

[7] Zhang F, Wang S, Li B et al (2022) Intradiscal injection for the management of low back pain. JOR Spine. 10.1002/jsp2.1186 10.1002/jsp2.1186 PMC8966879 35386759

[8] Veizi E, Hayek S (2014) Interventional therapies for chronic low back pain. Neuromodulation 17:31–45. 10.1111/ner.12250 25395115 10.1111/ner.12250

[9] Castromán P, Cristiani F, Surbano M et al (2019) Pulsed radiofrequency of the dorsal root ganglion for chronic lumbosacral radicular syndrome refractory to epidural steroid injections. Rev Soc Esp Dolor 26:166–174. 10.20986/resed.2019.3702/2018

[10] Wang D (2018) Image guidance technologies for interventional pain procedures: ultrasound, fluoroscopy, and CT. Curr Pain Headache Rep. 10.1007/s11916-018-0660-1 10.1007/s11916-018-0660-1 29374352

[11] Patel VB, Wasserman R, Imani F (2015) Interventional therapies for chronic low back pain: a focused review (efficacy and outcomes). Anesthesiol Pain Med. 10.5812/aapm.29716 10.5812/aapm.29716 PMC4604560 26484298

[12] Ding Y, Li H, Zhu Y et al (2018) Transforaminal epidural steroid injection combined with pulsed radio frequency on spinal nerve root for the treatment of lumbar disc herniation. J Pain Res 11:1531–1539. 10.2147/JPR.S174318 30147357 10.2147/JPR.S174318 PMC6097521

[13] Adıgüzel E, Tecer D, Güzelküçük Ü, Taşkaynatan MA, Tan AK (2017) The effectiveness of transforaminal epidural steroid injection in patients with radicular low back pain: combination of pain provocation with effectiveness results. Turk J Phys Med Rehabil 63:117–123. 10.5606/tftrd.2017.05882 31453439 10.5606/tftrd.2017.05882 PMC6648123

[14] Gray CM, Skinner C, Vasilopoulos T et al (2023) The Kumar technique: a novel and effective approach to transforaminal epidural steroid injections. Cureus. 10.7759/cureus.47210 10.7759/cureus.47210 PMC10653120 38022188

[15] Wei WB, Dang SJ, Wei L et al (2021) Transforaminal epidural steroid injection combined with radio frequency for the treatment of lumbar disc herniation: a 2-year follow-up. BMC Musculoskelet Disord. 10.1186/s12891-021-04209-5 10.1186/s12891-021-04209-5 PMC8042724 33845819

[16] Vanneste T, Van Lantschoot A, Van Boxem K, Van Zundert J (2017) Pulsed radiofrequency in chronic pain. Curr Opin Anaesthesiol. 10.1097/ACO.0000000000000502 10.1097/ACO.0000000000000502 28700369

[17] Kinci A, Sari S, Ertilav E, Aydin ON (2023) Efficacy of combined dorsal root ganglion pulsed radiofrequency and cervical epidural steroid injection on radicular neck pain. Turk Neurosurg 33:58–62. 10.5137/1019-5149.JTN.36295-21.3 35929031 10.5137/1019-5149.JTN.36295-21.3

[18] Munjupong S, Tontisirin N, Finlayson RJ (2016) The effect of pulsed radiofrequency combined with a transforaminal epidural steroid injection on chronic lumbar radicular pain: a randomized controlled trial. Adv Anesthesiol 2016:1–5. 10.1155/2016/2136381

[19] von Elm E, Altman DG, Egger M et al (2008) The Strengthening the Reporting of Observational Studies in Epidemiology (STROBE) statement: guidelines for reporting observational studies. J Clin Epidemiol 61:344–349. 10.1016/j.jclinepi.2007.11.008 18313558 10.1016/j.jclinepi.2007.11.008

[20] World Medical Association (2013) World Medical Association Declaration of Helsinki. JAMA 310:2191. 10.1001/jama.2013.281053 24141714 10.1001/jama.2013.281053

[21] Urden LD (2002) Patient satisfaction measurement: current issues and implications. Lippincotts Case Manag 7:194–200. 10.1097/00129234-200209000-00006 12394558 10.1097/00129234-200209000-00006

[22] Manchikanti L, Singh V, Cash KA et al (2012) Effect of fluoroscopically guided caudal epidural steroid or local anesthetic injections in the treatment of lumbar disc herniation and radiculitis: a randomized, controlled, double blind trial with a two-year follow-up. Pain Phys. 10.36076/ppj.2012/15/273 22828681

[23] Rathod DJ, Vora DD, Prajapati DD (2022) Study of outcome of fluoroscopic guided transforaminal lumbar epidural steroid injection in patients with low back pain with radiculopathy. Natl J Clin Orthop 6:87–91. 10.33545/orthor.2022.v6.i1b.352

[24] Conger A, Cushman DM, Speckman RA et al (2020) The effectiveness of fluoroscopically guided cervical transforaminal epidural steroid injection for the treatment of radicular pain: a systematic review and meta-analysis. Pain Med 21:41–54. 10.1093/pm/pnz127 31181148 10.1093/pm/pnz127

[25] Falsafi M, Baghianimoghadam B, Bahrami-Freiduni M, Esmaeilnejad-Ganji SM (2021) Examining the accuracy of ultrasound-guided lumbar transforaminal injection controlled by fluoroscopic imaging in patients with lumbar radiculopathy: a modified technique. Turk Neurosurg 31:582–586. 10.5137/1019-5149.JTN.32660-20.1 33978216 10.5137/1019-5149.JTN.32660-20.1

[26] Silbergleit R, Mehta BA, Sanders WP, Talati SJ (2001) Imaging-guided injection techniques with fluoroscopy and CT for spinal pain management. Radiographics 21:927–942. 10.1148/radiographics.21.4.g01jl15927 11452067 10.1148/radiographics.21.4.g01jl15927

[27] Artner J, Cakir B, Reichel H, Lattig F (2012) Radiation dose reduction in CT-guided sacroiliac joint injections to levels of pulsed fluoroscopy: a comparative study with technical considerations. J Pain Res 5:265–279. 10.2147/JPR.S34429 23028237 10.2147/JPR.S34429 PMC3442745

[28] Galhom AE, al-Shatouri MA (2013) Efficacy of therapeutic fluoroscopy-guided lumbar spine interventional procedures. Clin Imaging 37:649–656. 10.1016/j.clinimag.2013.02.006 23660156 10.1016/j.clinimag.2013.02.006

[29] Key BM, Tutton SM, Scheidt MJ (2023) Cone-beam CT with enhanced needle guidance and augmented fluoroscopy overlay: applications in interventional radiology. AJR Am J Roentgenol 221:92–102. 10.2214/AJR.22.28712 37095661 10.2214/AJR.22.28712

